# Prevalence, Determinants and Wealth-Related Inequality of Anxiety and Depression Symptoms Among Reproductive-Aged Women (15–49 Years) in Nepal: An Analysis of Nationally Representative Nepal Demographic and Health Survey Data 2022

**DOI:** 10.1155/da/9942669

**Published:** 2025-03-04

**Authors:** Syed Toukir Ahmed Noor, Samin Yeasar, Sazid Siddique, Rajon Banik, Sahar Raza

**Affiliations:** ^1^Maternal and Child Health Division, International Centre for Diarrhoeal Disease Research, Bangladesh (icddr,b), Dhaka, Bangladesh; ^2^Department of Statistics, Shahjalal University of Science and Technology, Sylhet 3114, Bangladesh

**Keywords:** anxiety, depression, mental health, Nepal, reproductive age, socio-economic inequality, women's health

## Abstract

**Background:** Mental health issues, particularly anxiety and depression, among women of reproductive age, remain a significant public health concern, yet comprehensive studies addressing these issues are limited in Nepal.

**Objective:** This study aimed to assess the prevalence, associated factors and wealth-related inequality of anxiety and depression symptoms among women aged 15–49 years in Nepal.

**Methods:** Data from the Nepal Demographic and Health Survey (NDHS) 2022 included 7410 women. Logistic regression analysis was conducted to identify factors associated with anxiety, depression and combined symptoms. We also employed the concentration curve to assess wealth-related disparities with mental health outcomes.

**Findings:** The prevalence of depression and anxiety symptoms was 5.4% (95% CI: 4.8% to 6.2%) and 7.5% (95% CI: 6.7% to 8.4%), respectively. Furthermore, 9.1% (95% CI: 8.2% to 10.1%) of the women experienced either condition, while 3.8% (95% CI: 3.3% to 4.4%) experienced both. Women with lower educational attainment, a higher number of children, unemployed partners, residents in rural areas and those living in Karnali province exhibited a higher prevalence of anxiety and depression symptoms. Women with limited mass media exposure were associated with a higher risk of anxiety and depression symptoms. Additionally, women with tobacco habits demonstrated a significantly higher risk of depression. Finally, wealth-related inequality was evident; women with lower socioeconomic status were more vulnerable to anxiety and depression symptoms.

**Conclusions:** The study highlights the need for targeted interventions addressing socio-economic determinants and lifestyle factors, including tobacco use, to mitigate the burden of anxiety and depression symptoms among women in Nepal.

**Summary**

• Anxiety and depression symptoms were prevalent among 7.5% and 5.4% of Nepalese women aged 15–49 years.
• Significant mental health disparities were found based on occupation, education and media exposure.
• Tobacco users showed higher odds of anxiety and depression symptoms.
• Wealth-related inequality was evident, with poorer women disproportionately affected.

## 1. Introduction

Mental health is a complex and multifaceted concept encompassing cognitive, emotional, and social well-being, playing a vital role in individuals' ability to respond to stress, form relationships and make decisions [1]. Anxiety and depression disorders are the two most common mental diseases worldwide among all the problems connected to mental health [2, 3]. Anxiety is characterised by excessive worry or fear about future events, while depression is marked by persistent sadness, hopelessness, and loss of interest in activities [4, 5]. Mental health is a critical component of the sustainable development goals, reflecting its growing recognition as a global health priority [6].

Globally, one in eight individuals experiences mental health issues, with anxiety disorders affecting 4% and depression affecting 5% of the population [3]. The burden of mental health issues is particularly significant among women of reproductive age, with prevalence rates increasing in low- and middle-income countries (LMICs) [7, 8]. Globally, mental health disorders are rising, with the number of affected individuals increasing from 654.8 million in 1990 to 970.1 million in 2019 (48.1% rise). This burden is especially pronounced among women, with a 50.1% rise in cases during this period [9]. Furthermore, studies from the USA, Spain, and Indonesia highlight the higher prevalence of anxiety and depression among women compared to men, with socioeconomic challenges increasing the likelihood of these disorders [10–12].

Furthermore, previous studies have identified various socio-demographic, behavioural and contextual factors associated with anxiety and depression among women, including older age, lower education, being unmarried, unemployment, low income, and psychosocial stress [12, 13]. In South Asia, socio-cultural and economic challenges such as lower educational levels, widowhood, lower socioeconomic status, joint family living arrangements, gender-based violence, early marriage and patriarchal norms exacerbate mental health risks for women [14, 15]. Other risk factors include intimate partner violence, low social support and gynaecological conditions [13, 16]. Despite their burden, a substantial proportion of mental health conditions among reproductive-aged women remain untreated [12].

Moreover, the prevalence of depression and anxiety symptoms is notably higher among women, reported at 22.1% and 28.8%, respectively, compared to 11.2% and 16.4% among men [17]. Projections from the Global Burden of Disease (GBD) study estimate that by 2050, depressive disorders will become the 7th leading cause of disease burden in Nepal, with anxiety disorders ranking 20th [18]. Despite this substantial burden, Nepal's health sector allocates less than 3% of the national budget to healthcare, with less than 1% dedicated to mental health services [19].

While some studies have explored anxiety and depression symptoms among Nepali women, most have been small-scale, regionally focused, and lacked national representation [20–22]. Women of reproductive age in Nepal face unique challenges, including gender discrimination, limited decision-making autonomy, and restricted access to resources and healthcare [23–25]. These systemic barriers contribute to the mental health challenges faced by Nepali women. To date, no nationally representative study has examined the prevalence, determinants and wealth-related inequality of anxiety and depression symptoms among women of reproductive age in Nepal. However, the Nepal Demographic and Health Survey (NDHS) 2022 has taken a significant step forward by including data on mental health in its most recent round and adding a dedicated section in the published report [26]. This represents a critical opportunity to better understand and address mental health outcomes among reproductive-aged women at a national level.

## 2. Objective

Therefore, the objective of this study is to estimate the prevalence of anxiety and depression symptoms among reproductive-aged women (15–49 years) in Nepal and to examine their significant determinants and wealth-related inequalities.

## 3. Methods

### 3.1. Data Source and Sampling Technique

For this research, we utilised data from the NDHS, a nationally representative cross-sectional survey carried out between 5 January and 22 June 2022 [26].

The National Statistical Office supplied a revised sample frame for the NDHS 2022 that was taken from the 2011 Nepal Population and Housing Census (NPHC). The census frame included Nepal's 36,020 sub-wards, categorised by urban or rural residence and household count. Nepal is now divided into seven provinces, districts, municipalities and wards as a result of administrative reforms made in 2015 that categorised urban and rural regions.

The 2022 NDHS used a stratified, two-stage sampling design to ensure representativeness across urban and rural areas as well as the seven provinces of Nepal. In the first stage, 476 primary sampling units (PSUs) were selected, with 248 PSUs drawn from urban areas and 228 from rural areas. These PSUs were chosen using probability proportional to size (PPS), meaning the probability of selection was based on the number of households in each PSU. After a household listing operation was conducted in each selected PSU, a total of 14,280 households were selected for the survey. In the second stage of sampling, 30 households were systematically selected from each PSU. Among these 14,280 selected households, half (7140 households) were randomly assigned to receive a more comprehensive questionnaire, which included additional modules on mental health and domestic violence. This more detailed questionnaire was administered to both men and women of those households. The remaining 7140 households were given a shorter version of the questionnaire, focusing on core demographic and health topics and interviewed only women aged 15–49 years of the households.

After household listing, A response rate of 99.7% was obtained from 13,786 out of 14,243 surveyed households. Of 15,238 eligible women (age 15–49), 14,845 were interviewed (response rate 97%). For eligible men (age 15–49), 4913 out of 5185 were interviewed (response rate 95%). Consequently, our analysis focuses on a subset of 7410 (weighted) women of reproductive age who participated in the more detailed survey, including the mental health module.

### 3.2. Study Variables and Measurements

#### 3.2.1. Outcome Variables

The Patient Health Questionnaire (PHQ-9) was used to assess depression in women between the ages of 15 and 49. The PHQ-9 is a popular self-report instrument made up of nine items that evaluate whether or not depressed symptoms have been present over the last 2 weeks [27]. Higher scores indicate more severe symptoms. The cumulative score, ranging from 0 to 27, delineates the following severity levels: mild (scores 5–9), moderate (scores 10–14), moderately severe (scores 15–19), severe (scores 20–27) and no depression (scores 0–4). In this study, a score of 10 or above indicated the existence of depressive symptoms (coded as 1), whereas a score of less than 10 denotes the absence of depressive symptoms (coded as 0) [27]. The Cronbach's alpha value of the PHQ-9 scale in our study was 0.864, indicating strong internal consistency.

Additionally, to assess anxiety in women between the ages of 15 and 49, the Generalised Anxiety Disorder Scale (GAD-7) was used. It is another self-report tool is the GAD-7, consisting of seven items that measure the occurrence and intensity of generalised anxiety symptoms during the previous 2 weeks [28]. The total score can be between 0 and 21. Similar to depression, anxiety symptoms were classified as present (coded as 1) for scores on the GAD-7 equal to or more than 10 and absent (coded as 0) for scores below 10 [28]. This scale resulted in a Cronbach's alpha coefficient of 0.857, which suggests strong instrument dependability. Frequency distributions for PHQ-9 and GAD-7 items are given in Table S1.

#### 3.2.2. Explanatory Variables

In this study, we utilised several explanatory variables covering socio-economic and demographic factors, maternal and paternal characteristics, household attributes and regional characteristics.

In terms of socio-economic and demographic factors, we considered women's age (15–19, 20–24, 25−29, 30–34, 35−39, 40–44 and 45−49), their education and husband/partners' education level (no education, primary, secondary, higher secondary or above), occupation (housewife, agriculture, professional/technical/managerial and clerical, skilled manual and others), husband/partners' age (15–19, 20–24, 25−29, 30–34, 35−39, 40–44, 45−49 and 50+), husband/partners' occupation (jobless, agriculture, professional/technical/managerial and clerical, skilled manual and others), status of marriage (never married and ever married) and religion (Hindu and others). Household attributes comprised the wealth index (poorest, poorer, middle, richer and richest), household head's sex (male and female), number of household members (≥4, 4>), the no. of children (0, 1, 2, 3 and 4+), currently residing with husband/partner (living with her and staying elsewhere), mass media exposure (no and yes), ownership of a mobile phone (no and yes). Additionally, we considered tobacco habit (no and yes), current pregnancy status (no and yes) and current breastfeeding status (no and yes). Division and area of residence were considered as regional-level variables, with residence categorised as (urban or rural).

### 3.3. Statistical Analysis

In this study, Stata version 17.0 (StataCorp LP, College Station, Texas) was used to clean, recode and analyse data in accordance with DHS guidance 7 [29]. To ensure the survey's representativeness, we used sample weights for weighting and made adjustments to the complex survey design by taking the PSU and strata into account. The Stata 'Svyset' command was also utilised to handle the complex survey design. Additionally, we followed the STROBE cross-sectional reporting guidelines [30].

We employed descriptive statistics to summarise the characteristics of the study sample. This involved calculating frequencies and row percentages for categorical variables, providing an overview of the distribution of each variable within the dataset. After that, the chi-squared test was performed to identify the relationship between the categorical factors and the outcome variable (anxiety and depression symptoms). Also, we used a multiple logistic regression analysis to find the variables linked to depression and anxiety among women aged 15–49 years. This study assessed multicollinearity using the variance inflation factor (VIF). The highest VIF found for the depression and anxiety models was 3.24 for the no. of children variable, and the mean VIF was 1.53. Due to multicollinearity, we omitted husband/partner-related variables (age, education, occupations, etc.) from the manuscript to ensure the robustness of our model. Every variable in any bivariate analysis with a *p*-value of less than 0.20 was included in our multivariable model. The adjusted odds ratio (AOR) with a 95% confidence interval (CI) was used to demonstrate the strength of the relationship.

### 3.4. Wealth-Related Inequality

Wealth-related inequality in this study refers to disparities in health outcomes based on the economic status of households, as measured by the DHS wealth index. This index categorises households into five quintiles-poorest, poorer, middle, richer and richest based on a composite score derived from household assets, living conditions and access to essential services [31]. The DHS wealth index offers a multidimensional measure of household wealth as an alternative to income alone, enabling a more nuanced evaluation of how economic status affects mental health outcomes [31].

In this study, we evaluated wealth-related inequality and its effects on mental health outcomes using the concentration curve (CC). The CC provided valuable insights into the impact of economic status on mental health outcomes among women of reproductive age by allowing us to see the distribution of anxiety and depression symptoms across different wealth groups [32]. When the concentration Index (CIX) is positive, it implies that mental health outcomes are more prevalent among wealthy people, and when it is negative, it shows that mental health problems are more prevalent in people with lower socioe-conomic levels. With 0 denoting an equal distribution among socio-economic groups, the CIX was computed using the ‘convenient covariance' method suggested by O'Donnell and colleagues [33]. In Stata ‘lorenz' [34] and ‘conindex' [35], commands were used to compute the concentration curve and CIX, respectively.

## 4. Results

**Figure 1:**
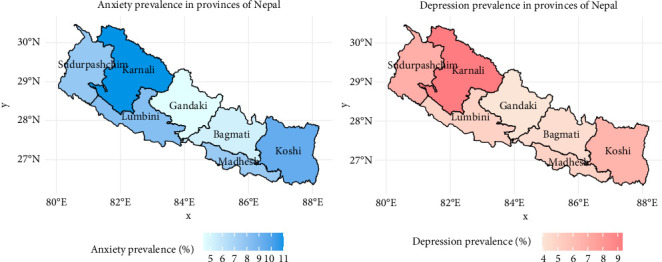
Prevalence of anxiety and depression symptoms among women aged 15–49 years by provinces of Nepal (*n* = 7410).

In this study, among the 7410 women surveyed (Table 1), 7.5% (95% CI: 6.7–8.4) reported experiencing anxiety, 5.4% (95% CI: 4.8–6.2) reported depression, 9.1% (95% CI: 8.2–10.1) reported either anxiety or depression and 3.8% (95% CI: 3.3–4.4) reported experiencing both conditions simultaneously.  Table 2 presents the distribution of various study variables among women aged 15–49 years, categorised by anxiety and depression symptoms. For the age of respondents, it was evident that anxiety and depression symptoms generally increase with age, peaking among women aged 45–49 years (anxiety: 7.9%, depression: 4.8%). In contrast, for the education of respondents, an inverse relation was found, women with no education (anxiety: 9.7%, depression: 6.3%) had the highest mental health outcomes compared to women with higher secondary or above education (anxiety: 2.5%, depression: 1.6%). Also, the husband/partner's education showed a similar pattern with mental health outcomes as respondents' education. Regarding occupation, women who did other work, like unskilled workers, showed the highest percentage of anxiety (13%) and depression (10.6%). Furthermore, women with unemployed husbands/partners had the highest percentage of mental health outcomes (anxiety: 14.5%, depression: 10.6%). Ever-married women showed a higher prevalence of anxiety and depression symptoms, with 8.3% reporting anxiety and 5.9% reporting depression. Regarding the wealth index, women from the poorest and poorer wealth quintiles exhibited elevated rates, with 7.9% and 9.9% reporting anxiety, respectively, and 6.4% and 6.3% reporting depression, respectively. Lack of mass media exposure was also associated with a higher prevalence of anxiety and depression symptoms, with 8.52% reporting anxiety and 6.25% reporting depression among women with no exposure. In terms of tobacco habit, the prevalence of mental health outcomes was notably higher among women who reported a tobacco habit compared to those who did not. Specifically, women who used tobacco showed a significantly higher prevalence of anxiety (11.35%) and depression (9.37%) compared to non-users. Regarding regional variation, urban women generally exhibited a lower prevalence than their rural counterparts, with provinces like Karnali showing the highest prevalence and Gandaki having the lowest (Figure 1).

**Figure 2:**
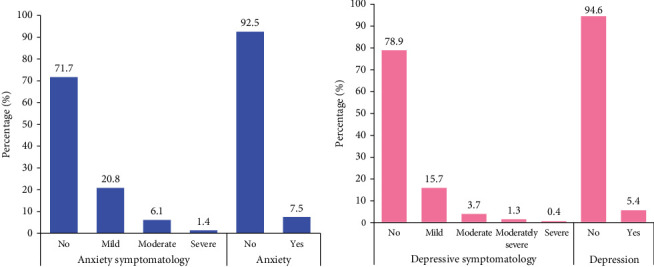
Prevalence and distribution of (a) anxiety and (b) depression symptoms among women aged 15–49 years (*n* = 7410).

Figure 2 displays the distribution of anxiety and depression symptoms at various levels among reproductive-aged women. For anxiety, 71.7% were found to have no symptoms, 20.8% exhibited mild symptoms, 6.1% had moderate symptoms and 1.4% experienced severe symptoms. Regarding depression, 78.9% had no symptoms, 15.7% exhibited mild symptoms, 3.7% had moderate symptoms, 1.3% experienced moderately severe symptoms and 0.4% had severe symptoms.

Table 3 represents the associated factors of mental health outcomes among reproductive-aged women in Nepal, yielding several significant findings. In terms of education, women with higher secondary education or above displayed a significantly reduced risk of anxiety symptoms (AOR = 0.39, 95% CI = 0.15, 0.99). Moving to occupation, women with other occupations (unskilled workers) had almost two times higher risk of anxiety and depression symptoms compared to housewives. Marital status revealed significant associations, with ever-married women displaying a substantially higher risk of anxiety symptoms (AOR = 2.90, 95% CI = 1.68, 4.99) and depression (AOR = 3.02, 95% CI = 1.54, 5.56). Transitioning to the wealth index, 'Poorer' individuals exhibited heightened odds of anxiety (AOR = 1.42, 95% CI = 1.16, 2.16). It was clear that women with tobacco habits had a 48% higher likelihood of depression (AOR = 1.48, 95% CI = 1.05, 2.08).

**Figure 3:**
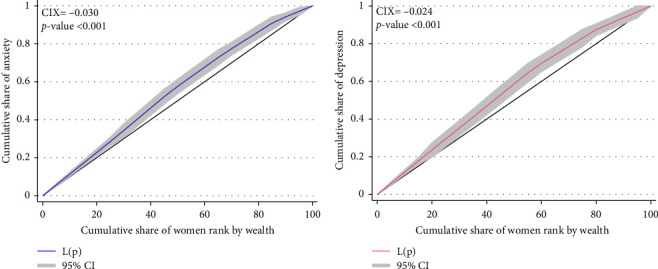
Concentration curve for (a) anxiety and (b) depression symptoms against wealth index among women aged 15–49 years (*n* = 7410).

Our study's results also showed a significant difference in the outcomes related to mental health concerning the wealth index among women in Nepal, as illustrated in Figure 3. This disparity is highlighted by the concentration curve, which consistently lies above the equality line for both anxiety and depression symptoms. Specifically, the CIX values further underscore this pattern, with CIX = −0.030 (*p*-value <0.001) for anxiety and CIX = −0.024 (*p*-value <0.001) for depression. These negative CIX values suggest that mental health issues, represented by anxiety and depression symptoms, were more concentrated among women with lower socio-economic status. In essence, women from poorer economic backgrounds are disproportionately affected by anxiety and depression symptoms compared to their wealthier counterparts.

## 5. Discussion

In this study, our primary objective was to comprehensively analyse the mental health status, specifically focusing on depression and anxiety among women of reproductive age in Nepal. The findings of this study shed light on the prevalence, determinants and wealth-related inequality of anxiety and depression symptoms in this demographic. The findings revealed the prevalence of mental health outcomes in this population, with 7.5% experiencing anxiety, 5.4% experiencing depression and 9.1% experiencing either anxiety or depression. Notably, 3.8% reported experiencing both anxiety and depression symptoms concurrently. The prevalence of depression observed in our study closely aligned with the GBD reported worldwide estimate for women of reproductive age, which stands at 5.2%. However, our study's anxiety prevalence was slightly elevated compared to the reported estimate of 5.9% [36]. Furthermore, our study also found that education level, occupation, marital status, wealth index, decision-making roles and tobacco habits were significantly associated factors of mental health outcomes. Additionally, our study identified a significant wealth-related inequality in mental health outcomes among women in Nepal.

The current study emphasises the significance of education in mental health outcomes by finding that greater levels of education were associated with a reduced risk of anxiety and anxiety/depression. The finding also aligns with several previous studies conducted in European countries and a study conducted in Bangladesh [37, 38]. Educated individuals may have better problem-solving skills, greater resilience and increased awareness of mental health issues, enabling them to effectively manage stressors and mitigate the risk of developing anxiety or depression [39, 40]. Expanding access to education, especially for women in rural areas, should be prioritised in Nepal to address these disparities. Policies such as the Nepal Education Sector Plan (2021–2030), which aims to improve literacy and access to education [41], should integrate mental health education as part of their framework to raise awareness and reduce stigma around mental health issues.

In our study, we found that the occupation of reproductive-aged women was significantly associated with anxiety and depression symptoms. Specifically, occupations like unskilled labour/domestic workers or retired personnel had a higher risk of anxiety and depression symptoms compared to housewives. In Nepal, women labourers experience job insecurity and low wages as they belong to unskilled workers [42]. As a result, job insecurity or low income may be the cause of anxiety and depression symptoms in women of reproductive age [43]. Furthermore, some other studies also revealed that occupations with lower skill levels or home maids had a higher risk of depression and anxiety [44, 45]. Similarly, another study found that retirement was associated with a higher risk of depression [46]. Nepal's Labour Act 2017 and Social Security Fund initiatives provide a framework for improving labour conditions [47]; however, these policies should be expanded to include mental health support programs, such as workplace counselling and mental health literacy campaigns, especially targeting women in vulnerable occupations.

The current study demonstrated that the sex of the household head was associated with depressive symptoms. In this instance of household head gender, women had a greater risk of depression compared to men. Specifically, female household heads face extra pressure and responsibilities in running the family and caring for other members [48]. Consequently, this extra pressure may result in an increased risk of depression among female household heads. Some similar papers also found that women household heads had a higher risk of depression compared to men [49, 50]. Addressing this issue in Nepal requires community-based support mechanisms for female household heads, including access to financial resources, counselling services and community outreach programs to alleviate their burdens.

Moreover, marital status also played a crucial role in determining mental health outcomes among women of reproductive age. Ever-married women showed a greater risk of anxiety and depression symptoms compared to their never-married counterparts. Several previous studies also found that married women were at a higher risk of anxiety and depression symptoms, with factors such as personal control, role demands and relationship problems playing a significant role [51, 52]. On the other hand, few other studies found the opposite result [53, 54]. The major reason behind this finding could be that marriage often brings about various life changes, including financial responsibilities, caregiving duties and relationship dynamics, which can increase stress levels. Additionally, societal expectations and traditional gender roles may place added pressure on married women to fulfil multiple roles, such as homemaker, caregiver and breadwinner, leading to heightened anxiety and depressive symptoms [55, 56]. This association may be attributed to various psychosocial factors inherent in marital relationships, including stressors related to matrimonial conflicts, caregiving responsibilities and social support dynamics [57, 58]. These findings highlight the importance of strengthening marital counselling services and mental health support systems for married women. Nepal's National Health Policy 2019 could incorporate strategies to provide marital and family counselling through primary healthcare centres, which would help reduce stressors related to marriage and caregiving.

The present study also found that tobacco habits were significantly associated with higher risks of depression and anxiety/depression for women of reproductive age. The same finding was evident in many previous studies that found tobacco smoking as a risk factor for depression [59–61]. Also, a systematic review revealed compelling evidence of a link between smoking and depression, indicating a 1.3 to 2.3-fold increase in depression prevalence among current smokers [62]. The main reason behind the observed link could be attributed to various factors, including the neurobiological effects of nicotine on the brain, the psychological impact of smoking as a coping mechanism and the social and environmental factors associated with smoking behaviour and mental health outcomes [62, 63]. Additionally, it is possible that individuals with depression may turn to smoking as a means of self-medication or stress relief, thereby exacerbating their depressive symptoms over time [62, 64]. Nepal's Tobacco Product (Control and Regulation) Act 2011 provides a foundation for reducing tobacco use [65]; however, targeted interventions for women, such as anti-tobacco campaigns addressing mental health risks, integrating smoking cessation programs into mental health services and stricter regulations are essential.

Additionally, geographical provinces were a significant factor in the determination of mental health outcomes among women of reproductive age. In this study, we found that the Madhesh Province, Bagmati Province and Gandaki Province had a lower risk of anxiety and anxiety/depression among women of reproductive age compared to Koshi Province. These findings may be attributed to stronger social networks, higher levels of urbanization and better socio-economic conditions in these provinces, which likely serve as protective factors against anxiety [66]. In addition, our study also revealed that the Karnali province had a greater risk of depression than the Koshi province. Karnali's high odds of depression may be explained by its severe socio-economic challenges, including widespread poverty, geographical isolation and limited access to healthcare and educational resources [67]. Furthermore, a nationwide study conducted in Nepal showed that the Madhesh Province had a significantly reduced frequency of lifetime mental disorders [68]. Policymakers in Nepal should focus on reducing regional disparities by improving healthcare infrastructure and implementing localised mental health programs in underserved areas such as Karnali. Strategies could include mobile mental health clinics, community-based psychosocial support programs and incentives to attract mental health professionals to remote areas.

Finally, the results of our study underscored a significant disparity in mental health outcomes among women in Nepal, particularly concerning wealth-related inequality. The concentration curve analysis further emphasised this disparity, as it consistently lay below the equality line for both anxiety and depression symptoms, indicating a disproportionate burden of mental health issues among women with lower wealth status (Figure 3). Our findings revealed that women from the poorest and poorer wealth quintiles exhibited elevated risk of anxiety and depression symptoms compared to their wealthier counterparts. This finding aligns with previous research conducted in Uganda [69], the US [70, 71] and New Zealand [72]. Targeted mental health interventions for economically disadvantaged people should be incorporated into Nepal's Multi-Sectoral Action Plan for Prevention and Control of Non-Communicable Diseases (2014–2020) in order to address these gaps. Subsidized mental healthcare, cash-transfer programs for families in poverty and community health worker initiatives could help alleviate the disproportionate burden of mental health issues among economically disadvantaged women.

### 5.1. Strength and limitations

Among the many strengths, the most important is that the study's statistical power and generalizability were increased by including a large, nationally representative sample size of 7410 women. Furthermore, the scope of the research and its particular focus on anxiety and depression symptoms among Nepalese women of reproductive age allows for a deeper understanding of the dynamics of mental health in this population. Moreover, examining wealth-related inequality through concentration curve analysis represents a novel contribution to the field, shedding light on wealth-related disparities in mental health outcomes.

Along with its strengths, the study has certain drawbacks that should be considered. First, the study's cross-sectional design limits the ability to establish causal linkages between variables, as data were collected at a single point in time. Additionally, the reliance on self-reported measures for symptoms of anxiety and depression introduces the possibility of response bias and underreporting. Future research would benefit from using structured interviews to assess clinically diagnosed mental health conditions, offering a more robust exploration of the relationship between sociocultural factors and mental disorders. Furthermore, the absence of data on variables such as physical illnesses, medications and substance use limits the study's ability to fully examine other contributing factors. We recommend that future primary research include these key variables for a more thorough investigation.

## 6. Conclusion

This study offers valuable insights into the prevalence, determinants and wealth-related inequalities of anxiety and depression symptoms among Nepalese women of reproductive age. The findings highlight several key factors associated with mental health outcomes, including education, employment, marital status, tobacco use and wealth inequality. Higher education levels were linked to lower odds of anxiety and depression, emphasising the importance of educational initiatives and awareness campaigns for mental health promotion. Policies should prioritise expanding access to education for women, particularly in rural and disadvantaged regions, as an essential step toward mitigating mental health disparities.

Wealth-related inequalities were evident, with women from lower socio-economic backgrounds experiencing a disproportionate burden of mental health symptoms. This underscores the need for targeted interventions that integrate mental health services into broader poverty alleviation programs, ensuring affordable and accessible mental healthcare for vulnerable populations. Tobacco use emerged as a strong factor, with smoking exacerbating mental health symptoms. Public health interventions, such as awareness campaigns highlighting the mental health risks of tobacco use and stricter regulatory measures, are crucial to reducing smoking prevalence among women. Geographical disparities further illustrated the uneven distribution of mental health risks, with provinces like Madhesh, Bagmati, and Gandaki exhibiting lower odds of anxiety and depression, likely due to stronger social networks and better socioeconomic conditions. In contrast, Karnali displayed a higher burden of depression, underlining the importance of addressing regional inequalities in healthcare access and social support. Localised mental health programs tailored to high-risk regions, such as Karnali, should improve healthcare infrastructure, expand access to mental health services, and strengthen community support systems.

In conclusion, policies focusing on expanding educational opportunities, reducing tobacco use, and addressing socio-economic and regional disparities could significantly mitigate the burden of mental health disorders among Nepalese women. Future research should continue to explore these factors, focusing on designing evidence-based strategies to reduce mental health disparities and improve the well-being of women in Nepal.

## Figures and Tables

**Table 1:** Distribution of study variables among women aged 15–49 years (*n* = 7410).

Variables	Total
	*n* (%)
Overall (weighted)	7410 (100)
Women age	
≤19	1322 (17.9)
20–24	1289 (17.4)
25–29	1230 (16.6)
30–34	1062 (14.3)
35–39	1005 (13.6)
40–44	804 (10.9)
45–49	698 (9.4)
Women education	
No education	1944 (26.2)
Primary	2256 (30.5)
Secondary	2931 (39.6)
Higher secondary or above	280 (3.8)
Husband/partners' age	
≤19	67 (1.2)
20–24	465 (8.4)
25–29	870 (15.7)
30–34	997 (18.0)
35–39	985 (17.8)
40–44	885 (16)
45–49	626 (11.3)
50+	636 (11.5)
Husband/partners' education	
No education	784 (14.4)
Primary	2225 (40.8)
Secondary	2065 (37.8)
Higher secondary or above	385 (7.1)
Respondents' occupation	
Housewife	2033 (27.4)
Agriculture	3591 (48.5)
Professional/technical/managerial and clerical	533 (7.2)
Skilled manual	903 (12.2)
Others	350 (4.7)
Husband/partner's occupation	
Jobless	138 (2.5)
Agriculture	1024 (18.6)
Professional/technical/managerial and clerical	727 (13.2)
Skilled manual	2485 (45.1)
Others	1131 (20.6)
Wealth index	
Poorest	1344 (18.1)
Poorer	1372 (18.5)
Middle	1512 (20.4)
Richer	1704 (23.0)
Richest	1480 (20.0)
Marital status	
Never married	1632 (22.0)
Ever married	5778 (78.0)
Religion	
Hindu	6151 (83.0)
Others	1259 (17.0)
No. of household members	
≥4	3471 (46.8)
4>	3940 (53.2)
Household heads' sex	
Male	4843 (65.4)
Female	2568 (34.7)
Mass media exposure	
No	3259 (44.0)
Yes	4151 (56.0)
Own a mobile phone	
No	1549 (20.9)
Yes	5861 (79.1)
Currently residing with husband/partner	
Living with her	3623 (65.5)
Staying elsewhere	1910 (34.5)
No. of child (ever born)	
0	2135 (28.8)
1	1316 (17.8)
2	1848 (24.9)
3	1086 (14.7)
4+	1026 (13.8)
Tobacco habit	
No	6852 (92.5)
Yes	558 (7.5)
Area of residence	
Urban	5064 (68.3)
Rural	2347 (31.7)
Province	
Koshi province	1241 (16.8)
Madhesh province	1512 (20.4)
Bagmati province	1493 (20.2)
Gandaki province	704 (9.5)
Lumbini province	1360 (18.4)
Karnali province	458 (6.2)
Sudurpashchim province	641 (8.7)

**Table 2:** Distribution of study variables by anxiety and depression symptoms among women aged 15–49 years (*n* = 7410).

Variables	Anxiety		Depression	
	Yes	*p*-Value	Yes	*p*-Value
	*N* (%)		*N* (%)	
Overall	555 (7.5)	—	403 (5.4)	—
Women age				
≤19	74 (5.6)	0.095	59 (4.5)	0.24
20–24	88 (6.8)		71 (5.5)	
25–29	99 (8.1)		68 (5.6)	
30–34	77 (7.3)		56 (5.3)	
35–39	84 (8.4)		54 (5.4)	
40–44	77 (9.5)		60 (7.5)	
45–49	55 (7.9)		33 (4.8)	
Women education				
No education	188 (9.7)	<0.001	122 (6.3)	<0.001
Primary	188 (8.3)		147 (6.5)	
Secondary	172 (5.9)		129 (4.4)	
Higher secondary or above	7 (2.5)		5 (1.6)	
Husband/partners' age				
≤19	6 (8.5)	0.516	2 (2.6)	0.582
20–24	31 (6.7)		27 (5.8)	
25–29	65 (7.4)		47 (5.4)	
30–34	78 (7.8)		61 (6.1)	
35–39	67 (6.8)		46 (4.7)	
40–44	67 (7.6)		44 (5)	
45–49	53 (8.5)		35 (5.6)	
50+	65 (10.3)		44 (6.9)	
Husband/partners' education				
No education	83 (10.6)	<0.001	48 (6.1)	0.018
Primary	201 (9)		142 (6.4)	
Secondary	126 (6.1)		100 (4.8)	
Higher secondary or above	12 (3.1)		11 (2.8)	
Respondents' occupation				
Housewife	114 (5.6)	0.003	88 (4.3)	0.001
Agriculture	305 (8.5)		213 (5.9)	
Professional/technical/managerial and clerical	33 (6.2)		25 (4.6)	
Skilled manual	57 (6.3)		40 (4.4)	
Others	45 (13)		37 (10.6)	
Husband/partner's occupation				
Jobless	20 (14.5)	0.002	15 (10.6)	0.022
Agriculture	88 (8.6)		59 (5.7)	
Professional/technical/managerial and clerical	35 (4.8)		23 (3.2)	
Skilled manual	187 (7.5)		141 (5.7)	
Others	101 (9)		63 (5.6)	
Wealth index				
Poorest	107 (7.9)	<0.001	86 (6.4)	0.006
Poorer	135 (9.9)		87 (6.3)	
Middle	120 (7.9)		96 (6.4)	
Richer	126 (7.4)		83 (4.9)	
Richest	68 (4.6)		50 (3.4)	
Marital status				
Never married	76 (4.7)	<0.001	61 (3.7)	0.007
Ever married	479 (8.3)		342 (5.9)	
Religion				
Hindu	466 (7.6)	0.578	323 (5.3)	0.144
Others	89 (7.1)		80 (6.4)	
No. of household members				
≥4	262 (7.6)	0.86	183 (5.3)	0.618
4>	293 (7.4)		220 (5.6)	
Household heads' sex				
Male	349 (7.2)	0.242	236 (4.9)	0.008
Female	206 (8)		166 (6.5)	
Mass media exposure				
No	278 (8.5)	0.014	204 (6.3)	0.015
Yes	277 (6.7)		199 (4.8)	
Own a mobile phone				
No	129 (8.4)	0.233	99 (6.4)	0.105
Yes	425 (7.3)		304 (5.2)	
Currently residing with husband/partner				
Living with her	275 (7.6)	0.471	193 (5.3)	0.392
Staying elsewhere	157 (8.2)		112 (5.9)	
No. of child (ever born)				
0	125 (5.9)	<0.001	100 (4.7)	0.003
1	83 (6.3)		62 (4.7)	
2	134 (7.3)		87 (4.7)	
3	104 (9.5)		74 (6.8)	
4+	109 (10.6)		79 (7.7)	
Tobacco habit				
No	491 (7.2)	<0.001	350 (5.1)	<0.001
Yes	63 (11.4)		52 (9.4)	
Area of residence				
Urban	358 (7.1)	0.144	252 (5)	0.046
Rural	196 (8.4)		151 (6.4)	
Province				
Koshi province	116 (9.4)	0.005	80 (6.5)	0.02
Madhesh province	115 (7.6)		76 (5)	
Bagmati province	79 (5.3)		65 (4.4)	
Gandaki province	32 (4.5)		28 (4)	
Lumbini province	113 (8.3)		67 (5)	
Karnali province	50 (11)		43 (9.3)	
Sudurpashchim province	49 (7.7)		43 (6.8)	

**Table 3:** Associated factors of anxiety and depression symptoms among reproductive-aged women in Nepal (*n* = 7410).

Variables	Anxiety		Depression	
	AOR (95% CI)	*p*-Value	AOR (95% CI)	*p*-Value
Women age				
≤19	Ref.	—	Ref.	—
20–24	1.00 (0.62,1.61)	0.994	1.09 (0.67,1.79)	0.726
25–29	1.09 (0.65,1.83)	0.732	1.07 (0.57,2.01)	0.832
30–34	0.87 (0.48,1.56)	0.634	0.92 (0.48,1.75)	0.797
35–39	0.85 (0.46,1.57)	0.605	0.79 (0.38,1.65)	0.534
40–44	0.89 (0.47,1.69)	0.713	1.04 (0.48,2.22)	0.928
45–49	0.70 (0.37,1.29)	0.249	0.59 (0.27,1.29)	0.186
Women education				
No education	Ref.	—	Ref.	—
Primary	0.99 (0.74,1.32)	0.935	1.32 (0.97,1.78)	0.073
Secondary	0.85 (0.60,1.20)	0.351	1.12 (0.79,1.60)	0.526
Higher secondary or above	0.39 (0.15,0.99)	0.049	0.48 (0.17,1.32)	0.154
Respondents' occupation				
Housewife	Ref.	—	Ref.	—
Agriculture	1.23 (0.9,1.68)	0.199	1.1 (0.82,1.49)	0.518
Professional/technical/managerial and clerical	1.52 (0.91,2.52)	0.109	1.43 (0.79,2.6)	0.24
Skilled manual	1.16 (0.75,1.78)	0.507	1.05 (0.6,1.83)	0.876
Others	2.03 (1.32,3.1)	0.001	1.99 (1.22,3.25)	0.006
Marital status				
Never married	Ref.	—	Ref.	—
Ever married	2.9 (1.68,4.99)	<0.001	3.02 (1.64,5.56)	<0.001
Religion				
Hindu	Ref.	—	Ref.	—
Others	0.91 (0.68,1.21)	0.527	1.27 (0.95,1.68)	0.105
Wealth index				
Poorest	Ref.	—	Ref.	—
Poorer	1.58 (1.16,2.16)	0.004	1.35 (0.96,1.9)	0.086
Middle	1.28 (0.91,1.81)	0.159	1.41 (0.97,2.05)	0.074
Richer	1.37 (0.98,1.93)	0.069	1.17 (0.79,1.74)	0.438
Richest	1.11 (0.7,1.75)	0.661	1.08 (0.62,1.89)	0.775
No. of household members				
≥4	Ref.	—	Ref.	—
4>	0.92 (0.73,1.15)	0.455	1.03 (0.77,1.36)	0.85
Household heads' sex				
Male	Ref.	—	Ref.	—
Female	1.08 (0.87,1.34)	0.506	1.36 (1.05,1.76)	0.021
Mass media exposure				
No	Ref.	—	Ref.	—
Yes	0.9 (0.71,1.15)	0.404	0.93 (0.73,1.19)	0.562
Own a mobile phone				
No	Ref.	—	Ref.	—
Yes	0.99 (0.74,1.32)	0.943	0.85 (0.62,1.15)	0.288
No. of child (ever born)				
0	Ref.	—	Ref.	—
1	0.74 (0.47,1.16)	0.19	0.81 (0.48,1.38)	0.438
2	0.81 (0.49,1.32)	0.39	0.83 (0.48,1.46)	0.52
3	1.03 (0.61,1.76)	0.905	1.18 (0.63,2.23)	0.599
4+	1.18 (0.66,2.11)	0.567	1.39 (0.73,2.64)	0.314
Tobacco habit				
No	Ref.	—	Ref.	—
Yes	1.3 (0.95,1.78)	0.098	1.48 (1.05,2.08)	0.026
Area of residence				
Urban	Ref.	—	Ref.	—
Rural	1.05 (0.81,1.36)	0.707	1.16 (0.87,1.54)	0.299
Province				
Koshi province	Ref.	—	Ref.	—
Madhesh province	0.63 (0.41,0.97)	0.036	0.63 (0.38,1.03)	0.063
Bagmati province	0.59 (0.38,0.92)	0.02	0.73 (0.43,1.23)	0.233
Gandaki province	0.47 (0.29,0.75)	0.002	0.64 (0.4,1.04)	0.071
Lumbini province	0.79 (0.54,1.18)	0.252	0.69 (0.44,1.09)	0.109
Karnali province	1.14 (0.76,1.72)	0.525	1.51 (1.04,2.21)	0.032
Sudurpashchim province	0.75 (0.5,1.14)	0.183	1.04 (0.68,1.6)	0.848

## Data Availability

Data are available on request from the DHS program website https://dhsprogram.com/data/dataset/Nepal_Standard-DHS_2022.cfm.

## Supporting Information

Supporting Information Supporting S1 provides a detailed breakdown of how often respondents experienced specific symptoms of depression and anxiety over the last 2 weeks, categorized as “Never,” “Rarely,” “Often,” or “Always.” Table S1. Frequency distributions for each item in the PHQ-9 and GAD-7 scales (*n* = 7410).
