# Supplementary material for: Prevalence, Determinants and Wealth-Related Inequality of Anxiety and Depression Symptoms Among Reproductive-Aged Women (15–49 Years) in Nepal: An Analysis of Nationally Representative Nepal Demographic and Health Survey Data 2022
Source: Depress Anxiety. 2025 Mar 4;2025:9942669. doi: 10.1155/da/9942669 (PMC11987074; doi:10.1155/da/9942669)
Supplement: Supporting Information — Supporting S1 provides a detailed breakdown of how often respondents experienced specific symptoms of depression and anxiety over the last 2 weeks, categorized as “Never,” “Rarely,” “Often,” or “Always.” Table S1. Frequency distributions for each item in the PHQ-9 and GAD-7 scales (n = 7410). [file 9942669.f1.docx]

**Supplementary**

Supplementary table 1: Frequency distributions for PHQ-9 and GAD-7 items (n=7410).

| Scale | Items | Never | Rarely | Often | Always |
| --- | --- | --- | --- | --- | --- |
|  |  | n (%) | n (%) | n (%) | n (%) |
| Over the last 2 weeks, how often have you been bothered by the following problems? | | | | | |
| GAD-7 items | 1. Feeling nervous, anxious or on edge | 3772 (50.91) | 2694 (36.36) | 667 (9) | 277 (3.74) |
|  | 2. Not being able to stop or control worrying | 5768 (77.84) | 1197 (16.16) | 333 (4.5) | 112 (1.51) |
|  | 3. Worrying too much about different things | 4098 (55.3) | 2446 (33.01) | 664 (8.96) | 202 (2.73) |
|  | 4. Trouble relaxing | 5130 (69.22) | 1744 (23.53) | 384 (5.18) | 153 (2.06) |
|  | 5. Being so restless that it is hard to sit still | 5816 (78.49) | 1198 (16.16) | 310 (4.18) | 87 (1.17) |
|  | 6. Becoming easily annoyed or irritated | 3252 (43.88) | 3148 (42.48) | 793 (10.7) | 217 (2.93) |
|  | 7. Feeling afraid as if something awful might happen | 4969 (67.06) | 1886 (25.45) | 445 (6) | 108 (1.46) |
|  |  |  |  |  |  |
| PHQ-9 items | 1. Little interest or pleasure in doing things | 5377 (72.56) | 1510 (20.38) | 416 (5.61) | 107 (1.45) |
|  | 2. Feeling down, depressed, or hopeless | 4900 (66.13) | 1934 (26.1) | 455 (6.14) | 122 (1.64) |
|  | 3. Trouble falling or staying asleep, or sleeping too much | 5135 (69.3) | 1732 (23.37) | 415 (5.59) | 129 (1.74) |
|  | 4. Feeling tired or having little energy | 4595 (62.01) | 2179 (29.4) | 515 (6.94) | 122 (1.64) |
|  | 5. Poor appetite or overeating | 5315 (71.72) | 1703 (22.99) | 319 (4.3) | 74 (1) |
|  | 6. Feeling bad about yourself - or that you are a failure or have let yourself or your family down | 6507 (87.81) | 679 (9.16) | 170 (2.29) | 54 (0.73) |
|  | 7. Trouble concentrating on things, such as reading the newspaper or watching television | 5893 (79.52) | 1206 (16.28) | 259 (3.49) | 53 (0.71) |
|  | 8. Moving or speaking so slowly that other people could have noticed? Or the opposite - being so fidgety or restless that you have been moving around a lot more than usual | 6608 (89.16) | 635 (8.57) | 134 (1.8) | 35 (0.47) |
|  | 9. Thoughts that you would be better off dead or of hurting yourself in some way | 6905 (93.18) | 392 (5.29) | 85 (1.15) | 28 (0.38) |
